# Evidence from Phylogenomics and Morphology Provide Insights into the Phylogeny, Plastome Evolution, and Taxonomy of *Kitagawia*

**DOI:** 10.3390/plants11233275

**Published:** 2022-11-28

**Authors:** Jia-Qing Lei, Chang-Kun Liu, Jing Cai, Megan Price, Song-Dong Zhou, Xing-Jin He

**Affiliations:** 1 Key Laboratory of Bio-Resources and Eco-Environment of Ministry of Education, College of Life Sciences, Sichuan University, Chengdu 610065, China; 2 Sichuan Key Laboratory of Conservation Biology on Endangered Wildlife, College of Life Sciences, Sichuan University, Chengdu 610065, China

**Keywords:** *Kitagawia*, *Peucedanum*, Apiaceae, morphology, phylogeny, plastome evolution, taxonomy

## Abstract

*Kitagawia* Pimenov is one of the segregate genera of *Peucedanum* sensu lato within the Apiaceae. The phylogenetic position and morphological delimitation of *Kitagawia* have been controversial. In this study, we used plastid genome (plastome) and nuclear ribosomal DNA (nrDNA) sequences to reconstruct the phylogeny of *Kitagawia*, along with comparative plastome and morphological analyses between *Kitagawia* and related taxa. The phylogenetic results identified that all examined *Kitagawia* species were divided into Subclade I and Subclade II within the tribe Selineae, and they were all distant from the representative members of *Peucedanum* sensu stricto. The plastomes of *Kitagawia* and related taxa showed visible differences in the LSC/IRa junction (JLA) and several hypervariable regions, which separated Subclade I and Subclade II from other taxa. Fruit anatomical and micromorphological characteristics, as well as general morphological characteristics, distinguished the four *Kitagawia* species within Subclade I from Subclade II and other related genera. This study supported the separation of *Kitagawia* from *Peucedanum* sensu lato, confirmed that *Kitagawia* belongs to Selineae, and two species (*K. praeruptora* and *K. formosana*) within Subclade II should be placed in a new genus. We believe that the “core” *Kitagawia* should be limited to Subclade I, and this genus can be distinguished by the association of a series of morphological characteristics. Overall, our study provides new insights into the phylogeny, plastome evolution, and taxonomy of *Kitagawia*.

## 1. Introduction

*Peucedanum* sensu lato, with 100–120 species distributed in Eurasia and Africa, is taxonomically one of the most complex groups in the Apiaceae [1]. *Peucedanum* sensu lato has long been regarded as extremely heterogeneous and contains a great diversity of life-forms, leaf and fruit structures, and chemical constituents [2,3]. Based on morphological and molecular studies, the genus is now reduced to only a few species allied to the type species *Peucedanum officinale* L., called *Peucedanum* sensu stricto, and several segregates are recognized as distinct genera [4,5,6,7,8,9].

*Kitagawia* Pimenov is one of the segregate genera of *Peucedanum* sensu lato. This genus was first described by the Russian botanist M. G. Pimenov in 1986 [10]. By investigating the carpological, morphological, and biochemical characteristics of species of *Peucedanum* sensu lato from the Far East and Siberia, Pimenov identified five species and one subspecies in the new genus *Kitagawia* and designated *Kitagawia terebinthacea* (Fisch. ex Trevir.) Pimenov as the nomenclatural type [10]. According to the original description of the genus by Pimenov [10], *Kitagawia* possesses distinguishing characteristics, such as partial lignification of mesocarp parenchyma and the absence of several flavonoids common to *Peucedanum* sensu lato.

However, the taxonomy of *Kitagawia* has long been controversial, and the boundaries of *Kitagawia* are poorly determined. Since the establishment of *Kitagawia*, many authors still include species treated as *Kitagawia* in *Peucedanum* sensu lato, and *Kitagawia* has been treated as a synonym of *Peucedanum* sensu lato [11,12,13,14]. In addition, several recently discovered Korean endemics that morphologically resemble *Kitagawia* species were also included in *Peucedanum* sensu lato [15,16]. Only a few botanists have thought it necessary to separate *Kitagawia* from *Peucedanum* sensu lato. Of them, Pimenov, the authority on *Kitagawia*, stated in 1986 that the boundaries of *Kitagawia* may expand further (“some uncovered species of *Peucedanum* sensu lato and *Angelica* in China and Japan should probably be transferred to *Kitagawia*”) [10]. Pimenov and Ostroumova [17] revised the description of the genus and included nine species in *Kitagawia*. Meanwhile, four species of *Peucedanum* sensu lato endemic in China, *Peucedanum formosanum* Hayata, *Peucedanum ampliatum* K.T. Fu, *Peucedanum harry-smithii* Fedde ex H. Wolff, and *Peucedanum songpanense* R.H. Shan & F.T. Pu, were considered as possible candidates for inclusion in *Kitagawia* [17]. In 2017, Pimenov identified five new nomenclatural combinations for *Kitagawia* by consulting the type specimens of Chinese Apiaceae [18]. Currently, ten species have been identified in *Kitagawia* [4]. Eight of these species, including the type species *K. terebinthacea*, are found in China.

The morphological delimitation of *Kitagawia* is ambiguous. In traditional taxonomy, fruit characteristics are of crucial importance in the classification system of the Apiaceae, and this is true for the identification of *Kitagawia* [10,19,20,21,22]. However, three newly identified *Kitagawia* species, *Kitagawia macilenta* (Franch.) Pimenov, *Kitagawia formosana* (Hayata) Pimenov, and *Kitagawia praeruptora* (Dunn) Pimenov have more than one vallecular vitta and more than two commissural vittae, and the latter two species have hairy mericarps, all incongruent with the original description of *Kitagawia* [10,11,12]. In addition, similar characteristics such as mesocarp cells with pitted walls, vallecular vittae one, and commissural vittae two are shared by *Kitagawia*, *Peucedanum* sensu stricto, and other segregate genera of *Peucedanum* sensu lato [4], which blur the morphological delimitations among them. Recently, fruit micromorphological studies performed by Ostroumova [23,24,25] have revealed a new diagnostic characteristic for *Kitagawia* (fruits are either pubescent or have areas with rugulate cuticles). Nevertheless, this characteristic was not reported in a similar study by Lee et al. [26]. Therefore, whether fruit micromorphological characteristics can provide support for the distinction of *Kitagawia* needs further examination.

The molecular phylogeny of *Kitagawia* is complicated and unresolved. *Kitagawia terebinthacea* (Fisch. ex Trevir.) Pimenov, the type species of *Kitagawia*, has shown different phylogenetic placements in several main clades (i.e., the tribe Selineae, Pleurospermeae, and *Acronema* Clade) in various phylogenetic analyses [9,27,28,29]. The bewildering results undoubtedly imply that some accessions of *Kitagawia* were misidentifications, as confirmed by Downie et al. [30]. The result of Downie et al. [30] that *Kitagawia* was divided into the tribe Selineae and *Acronema* Clade may also have been misleading. Recent phylogenetic studies conducted by Pimenov et al. suggested that *Kitagawia* was not closely related to *Peucedanum* sensu stricto and other segregates of *Peucedanum* sensu lato but clustered with *Saposhnikovia* Schischk., which to some extent supported the separation of *Kitagawia* [5,9,31]. However, some *Kitagawia* species belonging to Selineae did not cluster together, according to Zhou et al.’s [32,33] phylogenetic analyses. Thus, more reliable identification and more robust phylogenetic reconstruction are required for *Kitagawia*.

It is notable that the aforementioned phylogenetic analyses relied on single DNA fragments [e.g., nuclear ribosomal DNA internal transcribed spacer (nrDNA ITS) and chloroplast DNA (cpDNA) *rpl16* and *rps16* intron], and they suffered from low branch support. Plastids are important organelles in plants. In most angiosperms, including *Daucus* L. and *Foeniculum* Mill. in the Apiaceae, plastid genomes (plastomes) are predominantly maternally inherited [34,35]. Plastomes lack recombination and have low rates of nucleotide substitutions [36,37]. Adequate phylogenetic informative characters are another important advantage of plastomes. These characteristics have led to their widespread use in analyses of phylogenetic relationships [38,39,40]. Plastome sequences can effectively improve the support and resolution of phylogenies at the generic level and beyond [41,42,43,44,45,46]. In particular, the plastome-based phylogeny performed by Liu et al. [47] constructed a robust phylogenetic framework for *Peucedanum* sensu lato and provided a valuable reference to our investigation of *Kitagawia*. In addition, comparative analyses of plastomes can also provide useful information for eliciting evolutionary and interspecific relationships [48,49,50], which should further improve our understanding of the taxonomic classification of *Kitagawia*.

The combination of molecular data and morphological characteristics has proven to yield solid evidence for the phylogeny and taxonomy of many Apiaceae [51,52,53,54,55]. In the present study, we performed comprehensive phylogenetic analyses based on plastomes and nrDNA sequences, complemented by detailed comparative plastome and morphological analyses of *Kitagawia* and related taxa. The objectives of this study were to: (1) reconstruct the phylogeny of *Kitagawia*; (2) investigate the plastome features of *Kitagawia* and related taxa; (3) verify the taxonomic value of fruit micromorphology for *Kitagawia*; (4) examine previous taxonomic treatments and proposals for *Kitagawia*.

## 2. Results

### 2.1. Phylogenetic Analyses

**Figure 1:**
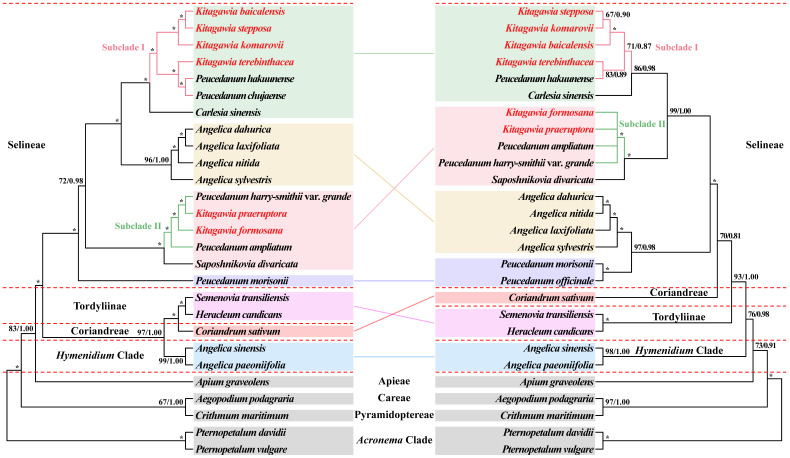
Phylogenetic trees constructed by maximum likelihood (ML) and Bayesian inference (BI). The bootstrap values (BS) of ML and posterior probabilities (PP) of BI are listed at each node. (*) represents maximum support in both analyses. Colored blocks indicate the main clades or branches with inconsistent phylogenetic placements in both phylogenetic trees. (**Left**): CDS tree; (**Right**): nrDNA (ITS + ETS) tree.

Single-copy coding sequences (CDS) and nrDNA (ITS + ETS) sequences were used to conduct the phylogenetic analyses. The phylogenetic trees based on the plastome CDS dataset and nrDNA dataset produced incongruent tree topologies, while they identified that all six *Kitagawia* species examined fell into Selineae but were not clustered in one branch (Figure 1). Two types of support values, maximum likelihood (ML) bootstrap values (BS) and Bayesian inference (BI) posterior probabilities (PP), are shown on the phylogenetic trees (Figure 1).

The analyses of ML and BI generated identical tree topologies for the plastome CDS dataset (Figure 1). In Selineae, *Kitagawia baicalensis* (Redow. ex Willd.) Pimenov, *Kitagawia stepposa* (Y.H. Huang) Pimenov, *Kitagawia komarovii* Pimenov, and *K. terebinthacea* clustered with *Peucedanum hakuunense* Nakai and *Peucedanum chujaense* K. Kim, S.H. Oh, Chan S. Kim & C.W. Park forming a robust branch, namely Subclade I (BS = 100%, PP = 1.00). *Kitagawia praeruptora* (Dunn) Pimenov, *K. formosana*, *P. harry-smithii* var. *grande* (K.T. Fu) R.H. Shan & M.G. Sheh, and *P. ampliatum* formed Subclade II with strong support (BS = 100%, PP = 1.00). Subclade I and Subclade II were sister to *Carlesia sinensis* Dunn and *Saposhnikovia divaricata* (Turcz.) Schischk. (BS = 100%, PP = 1.00), respectively. Within Subclade I, *K. baicalensis* was sister to *K. stepposa* (BS = 100%, PP = 1.00), then they were clustered with *K. komarovii* forming one branch of Subclade I (BS = 100%, PP = 1.00). *Peucedanum hakuunense* Nakai was more closely related to *P. chujaense* (BS = 100%, PP = 1.00), then clustered with *K. terebinthacea* forming another branch of Subclade I (BS = 100%, PP = 1.00). Within Subclade II, *P. ampliatum* first diverged from the remaining species (BS = 100%, PP = 1.00), followed by *K. formosana* (BS = 100%, PP = 1.00), and the branch *K. praeruptora* + *P. harry-smithii* var. *grande* (BS = 100%, PP = 1.00). The branch Subclade I + *C. sinensis* were sister to four *Angelica* species (BS = 100%, PP = 1.00); they were then clustered with the branch Subclade II + *S. divaricata* (BS = 72%, PP = 0.98). *Peucedanum morisonii* Besser ex Schult. was located at the base of Selineae (BS = 100%, PP = 1.00) and was distant from Subclade I and Subclade II.

The nrDNA (ITS + ETS) tree topologies generated by ML and BI analyses were also consistent (Figure 1). In Selineae, the branch Subclade I + *C. sinensis* were no longer sister to four *Angelica* species but were sister to the branch Subclade II + *S. divaricata* (BS = 100%, PP = 1.00). For Subclade I, *K. stepposa* was no longer sister to *K. baicalensis*, but formed a sister branch to *K. komarovii* (BS = 69%, PP = 0.94), then clustered with *K. baicalensis* (BS = 100%, PP = 1.00). *Peucedanum hakuunense* Nakai was sister to *K. terebinthacea* (BS = 85%, PP = 0.91), and they continued to cluster with *K. stepposa*, *K. komarovii*, and *K. baicalensis*, but with only moderate support (BS = 68%, PP = 0.87). Within Subclade II, *K. formosana*, *K. praeruptora*, *P. ampliatum*, and *P. harry-smithii* var. *grande* formed a polytomy with strong support (BS = 100%, PP = 1.00). *Peucedanum officinale* L. was sister to *P. morisonii* (BS = 100%, PP = 1.00), and they were clustered with four *Angelica* species (BS = 97%, PP = 0.99).

Three clades, Tordyliinae, Coriandreae, and *Hymenidium* Clade, had very different phylogenetic placements in the two phylogenetic trees (Figure 1). In the CDS tree, Tordyliinae was more closely related to Coriandreae (BS = 100%, PP = 1.00), and they were then clustered with *Hymenidium* Clade forming a branch with high support (BS = 97%, PP = 1.00). In the nrDNA (ITS + ETS) tree, the three clades were not clustered in one branch. *Hymenidium* Clade was first to diverge (BS = 76%, PP = 0.99), followed by Tordyliinae (BS = 97%, PP = 1.00), and a Coriandreae + Selineae branch (BS = 63%, PP = 0.77).

### 2.2. Comparative Analyses of Plastomes

#### 2.2.1. Plastome Features of Kitagawia and Related Taxa

The complete plastome sequences range in size from 146,718 bp (*K. formosana*) to 148,327 bp (*K. terebinthacea*) for the 14 species of *Kitagawia* and related taxa (Table 1, Figure S1). All these plastomes comprised a pair of inverted repeat (IR) regions (17,987–19,043 bp) separated by the large single copy (LSC, 91,829–93,700 bp) and small single copy (SSC) regions (17,377–17,631 bp), exhibiting a typical quadripartite structure [43,47] (Table 1, Figure S1). The IR regions of three species (17,987 bp for *P. morisonii*, 18,058 bp for *Angelica sylvestris* L., and 18,095 bp for *C. sinensis*) were the shortest in length (Table 1). The overall GC content was between 37.4–37.6%, with the IR regions richer (44.3–44.8%) than the LSC (35.9–36.1%) or SSC (30.8–31.1%) regions (Table 1, Figure S1). All 14 plastomes encoded 113–114 unique genes, with 80 protein-coding genes (PCGs), 29–30 transfer RNA (tRNA) genes, and 4 ribosomal RNA (rRNA) genes (Table 1 and Table S1). In comparison to other samples in this study, the plastomes of *K. praeruptora*, *K. formosana*, and *Peucedanum harry-smithii* var. *grande* lacked the *trnT-GGU* gene (Figure S1, Table S1). Fifteen genes were duplicated in the IR regions, including five PCGs, six tRNA genes, and four rRNA genes (Table S1). Incomplete copy of gene *ycf1* in the IRb region was regarded as a pseudogene (*ψycf1*) (Table S1). There were 16 intron-containing genes found, 13 of which were PCGs, and the remainder were tRNA genes (Table S1). Three genes (*ycf3*, *clpP*, and *rps12*) possessed two introns, whereas the others each contained only one (Table S1).

#### 2.2.2. Simple Sequence Repeat Analysis

**Figure 2:**
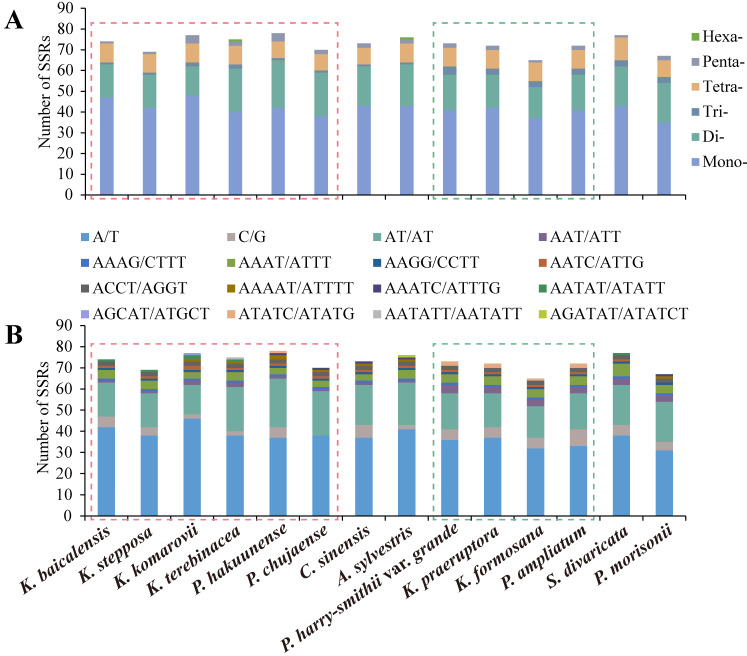
Comparison of simple sequence repeats (SSRs) in the 14 plastomes of *Kitagawia* and related taxa. (**A**) number of six types of SSRs; (**B**) number of all repeat motifs of identified SSRs. Boxes in pink and green are for Subclade I and Subclade II, respectively.

Among the 14 selected plastomes, the total number of simple sequence repeats (SSRs) ranged from 65 (*K. formosana*) to 78 (*P. hakuunense*) (Figure 2, Table S2). The number of mononucleotides (35–48) was the largest, followed by dinucleotides (14–23), tetranucleotides (8–11), and trinucleotides (1–4) (Figure 2, Table S2). Hexanucleotides were the least common, only found in *K. terebinthacea* and *A. sylvestris* (Figure 2, Table S2). In addition, bases A and T were the dominant components for all identified SSRs in the 14 plastomes (Figure 2, Table S2). In all plastomes, mononucleotides, dinucleotides, and trinucleotides were composed almost entirely of A and T, except for the C/G motif (0–11.11%) of mononucleotides (Figure 2, Table S2).

#### 2.2.3. IR Border Analysis

**Figure 3:**
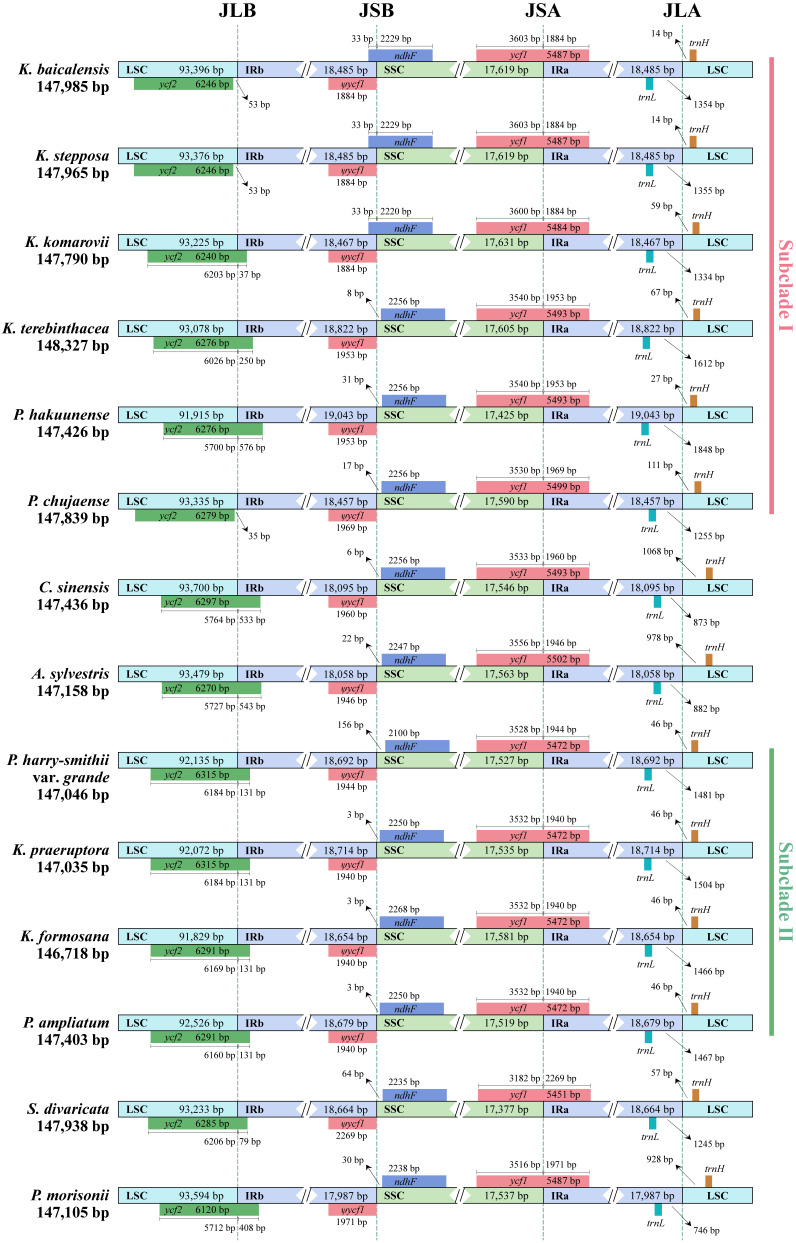
Comparison of the border regions of the 14 plastomes of *Kitagawia* and related taxa. Different boxes for genes represent the gene position.

The IR border regions and adjacent genes of the complete plastomes from 14 selected species of *Kitagawia* and related taxa were compared to analyze expansion and contraction in junction regions. Overall, Subclade I and Subclade II were similar at the four junctions, while both markedly differed from *P. morisonii*, *C. sinensis*, and *A. sylvestris*. The LSC/IRa junction (JLA) of the 14 plastomes occurred between genes *trnL* and *trnH*. There were 14–111 bp of non-coding sequence between JLA and the 3′ end of gene *trnH* in Subclade I and Subclade II, which were considerably less than 928 bp in *P. morisonii*, 1068 bp in *C. sinensis*, and 978 bp in *A. sylvestris* (Figure 3). Conversely, there were 1255–1848 bp of non-coding sequence in Subclade I and Subclade II between JLA and the 3′ end of gene *trnL*, which were much more than 746 bp in *P. morisonii*, 873 bp in *C. sinensis*, and 882 bp in *A. sylvestris* (Figure 3). The four junctions in Subclade II were relatively consistent, whereas, within Subclade I, they were more diverse. The LSC/IRb junction (JLB) was mostly located within gene *ycf2*, with 37–576 bp of gene *ycf2* duplicated in the IRb region, except that the distance from JLB to the 3′ end of gene *ycf2* was 35–53 bp in *K. baicalensis*, *K. stepposa*, and *P. hakuunense* in Subclade I (Figure 3). Similarly, the SSC/IRb junction (JSB) were mostly located between pseudogene *ψycf1* and gene *ndhF*, and were 3–156 bp away from the 3′ end of gene *ndhF*, except for *K. baicalensis*, *K. stepposa,* and *K. komarovii*, where gene *ndhF* spanned JSB, and 33 bp of gene *ndhF* were duplicated in the IRb region (Figure 3). In the 14 plastomes, gene *ycf1* spanned the SSC/IRa junction (JSA) (Figure 3). For *K. baicalensis*, *K. stepposa*, and *K. komarovii* in Subclade I, 1884 bp of gene *ycf1* duplicated in the IRa region, while for the remaining plastomes, the duplication portions of gene *ycf1* in the IRa region were longer (1953–2269 bp) (Figure 3).

#### 2.2.4. DNA Rearrangement and Sequence Divergence Analyses

**Figure 4:**
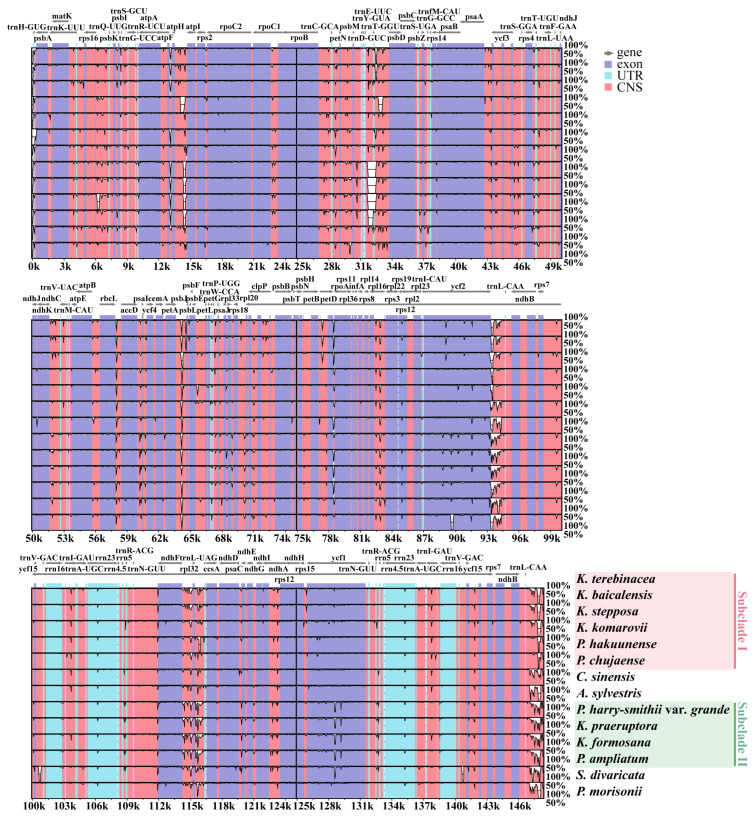
Sequence identity plot of the 14 plastomes with *K. terebinthacea* as the reference. The vertical scale represents the percentage of identity ranging from 50 to 100%. Coding and non-coding regions are marked in purple and pink, respectively.

The DNA arrangement of the 14 plastomes examined was relatively conserved. No gene rearrangements were detected among them (Figure S2). With reference to *K. terebinthacea*, the overall sequence identity and divergent regions across the 14 selected plastomes were analyzed. Clearly, the LSC and SSC regions were more divergent than the two IR regions, and coding regions showed more sequence conservation than non-coding regions (Figure 4). In several highly variable regions (e.g., *atpF-atpH*, *petN-psbM*, *accD-psaI*, *ycf2-trnL*, *rpl32-trnL*, *ycf1*, and *ycf2*), Subclade I showed a high degree of similarity, which differed from Subclade II and *P. morisonii* (Figure 4).

### 2.3. Morphological Analyses

#### 2.3.1. Fruit Anatomical and Micromorphological Examination

Fruit anatomical and micromorphological characteristics of the five *Kitagawia* species studied are shown in Table 2.

**Figure 5:**
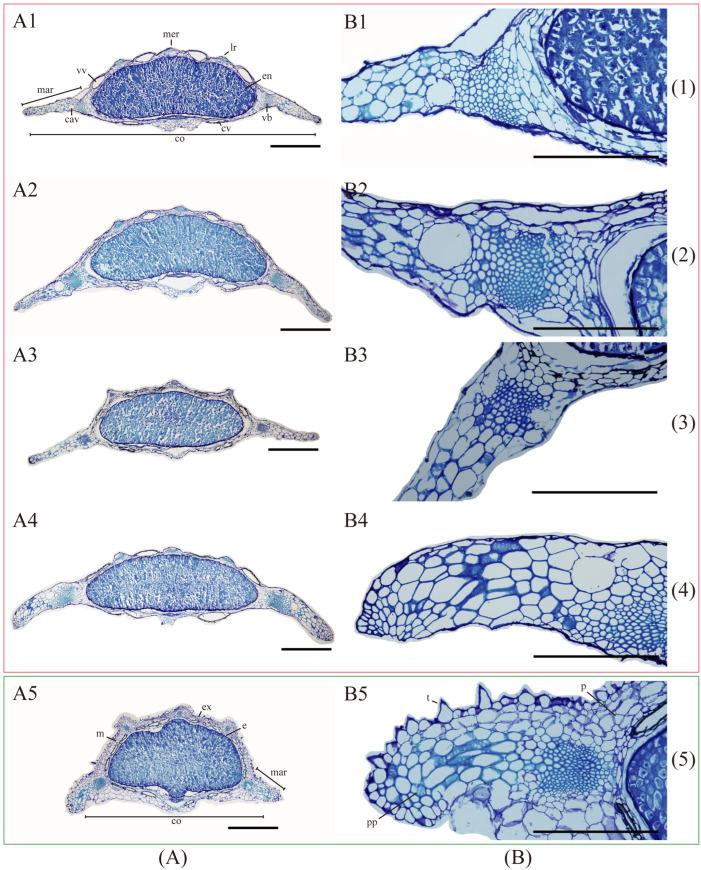
Fruit anatomical characteristics of five *Kitagawia* species. (**A**) transverse sections; (**B**) mesocarp parenchyma in marginal ribs. (**1**) *K. baicalensis*; (**2**) *K. stepposa*; (**3**) *K. komarovii*; (**4**) *K. terebinthacea*; (**5**) *K. praeruptora*. Abbreviations: cav = cavity; co = commissure; cv = commissural vittae; e = endocarp; en = endosperm; ex = exocarp; lr = lateral ribs; m = mesocarp; mar = marginal ribs; mer = median rib; p = parenchyma without pits; pp = lignified parenchyma with pitted walls; t = trichomes; vb = vallecular bundles; vv = vallecular vittae. Boxes in pink and green are for Subclade I and Subclade II, respectively. Scale bar = 0.5 mm in (**A**); 0.25 mm in (**B**).

Fruit anatomical characteristics showed that the four *Kitagawia* species within Subclade I (i.e., *K. baicalensis*, *K. komarovii*, *K. stepposa*, and *K. terebinthacea*) all had strongly dorsally compressed mericarps, filiform and slightly prominent dorsal (one median and two lateral) ribs, winged marginal ribs, flat or slightly concave endosperm commissural face, solitary vitta in each furrow, 2 vittae on commissure, 1–2 layers of mesocarp cells closest to the epidermis, and lignified parenchyma with pitted walls in mesocarp (Table 2, Figure 5). *Kitagawia praeruptora* (Dunn) Pimenov within Subclade II had slightly dorsally compressed mericarps, filiform and prominent dorsal ribs, narrowly winged and thick marginal ribs, flat endosperm commissural face, 3–4 vittae in each furrow, 6 vittae on commissure, 3–5 layers of mesocarp cells closest to the epidermis, and lignified parenchyma with pitted walls in mesocarp (Table 2, Figure 5). The average ratios of the total widths of both marginal ribs to the entire fruit width were 42.3–50.3% in the four *Kitagawia* species within Subclade I (*K. baicalensis*, *K. komarovii*, *K. stepposa*, and *K. terebinthacea*), while the average ratios of the commissure width to the fruit width were 94.1–96.4% (Table 2, Figure 5). In *K. praeruptora*, these two average ratios were 39.8% and 85.2%, respectively (Table 2, Figure 5).

**Figure 6:**
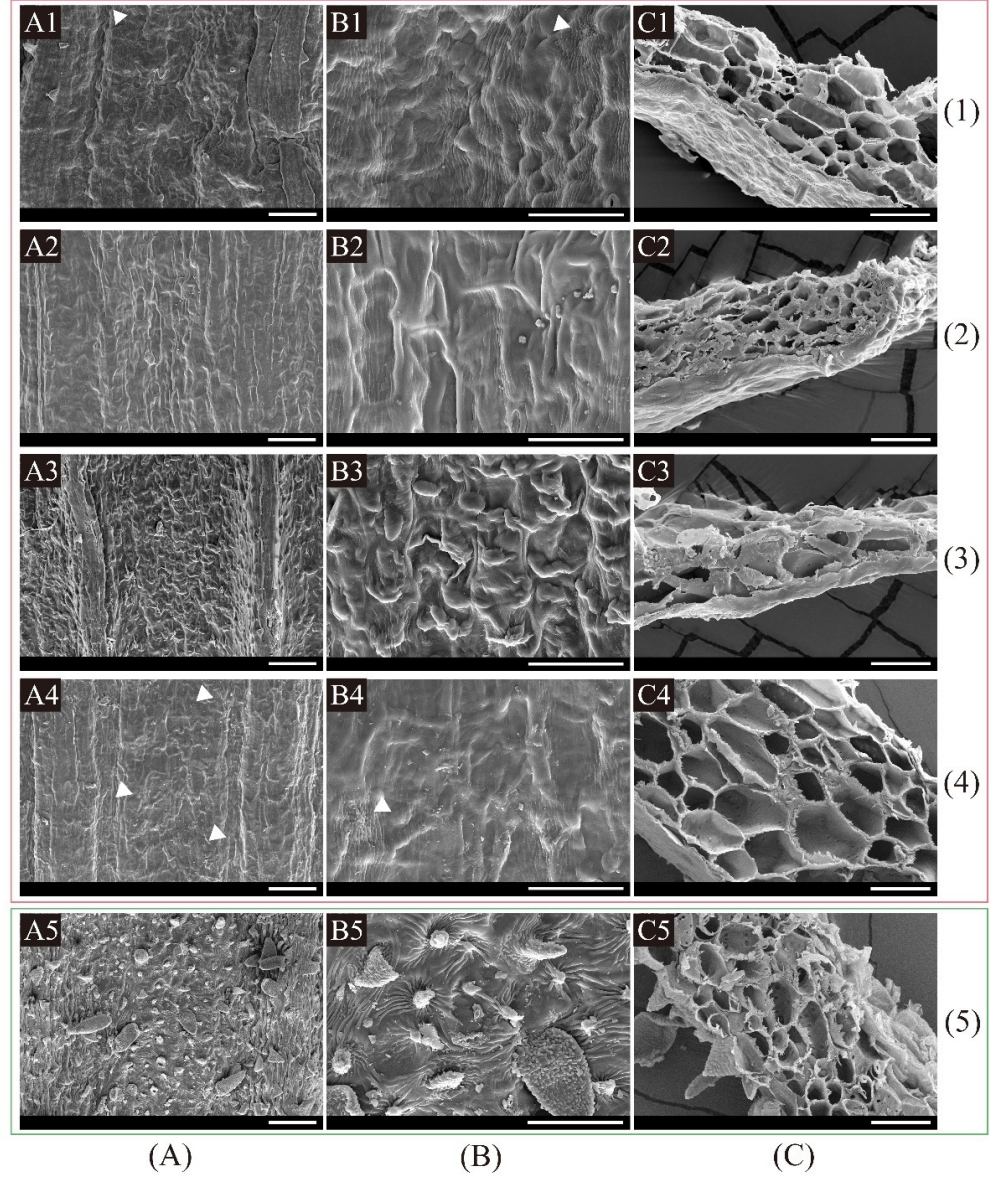
Fruit micromorphological characteristics of five *Kitagawia* species. (**A**) surface patterns of fruits; (**B**) cuticular foldings of fruit surfaces; (**C**) inner structure in marginal ribs of fruits. (**1**) *K. baicalensis*; (**2**) *K. stepposa*; (**3**) *K. komarovii*; (**4**) *K. terebinthacea*; (**5**) *K. praeruptora*. Rugulate cuticles are marked with white triangles. Boxes in pink and green are for Subclade I and Subclade II, respectively. Scale bar = 100 µm in (**A**); 50 µm in (**B**,**C**).

Fruit micromorphological studies revealed that the five *Kitagawia* species had inconspicuous cell borders of fruit surfaces, different fruit surfaces (undulate: *K. baicalensis* and *K. terebinthacea*; rugate: *K. stepposa*; tuberculate: *K. komarovii*; raised: *K. praeruptora*), various cuticular foldings (cuticle striate and rugulate: *K. baicanlensis* and *K. terebinthacea*; cuticle smooth and striate: *K. stepposa* and *K. komarovii*; dense, tiny prickles and short hairs with rugulate and tuberculate cuticle: *K. praeruptora*), without epicuticular wax, and parenchyma cells with pitted walls in marginal ribs (Table 2, Figure 6).

#### 2.3.2. Comparison of General Morphological Characteristics

Considering general morphological characteristics, the four *Kitagawia* species within Subclade I shared glabrous stems, 2–3-pinnate leaf blades, lanceolate bracts and bracteoles, unequal and adaxial surface hispid rays, white petals, subulate or triangular calyx teeth, conical stylopodia, and elliptic, glabrous mericarps (Table S3). *Kitagawia praeruptora* (Dunn) Pimenov and *K. formosana* within Subclade II shared hairy stems and rays, linear or lanceolate bracts and bracteoles, white petals, obsolete or inconspicuous calyx teeth, conical stylopodia, and ovate-elliptic, sparsely pubescent or hispid mericarps (Table S3). More morphological characteristics for all 15 species studied are summarized in Table S3.

## 3. Discussion

### 3.1. Phylogenetic Position and Non-Monophyly of Kitagawia

Previous phylogenetic analyses did not specifically examine the genus *Kitagawia* and may have been misled by misidentifications of some *Kitagawia* accessions, failing to identify a clear phylogenetic position for *Kitagawia* [27,29,30]. Recent studies have revealed that *Kitagawia* is distantly related to *Peucedanum* sensu stricto and other segregates of *Peucedanum* sensu lato, but they relied on single DNA fragments and were limited by low support [5,9,31]. In this study, we combined complete plastome sequences with nrDNA sequences to produce a well-supported phylogeny for *Kitagawia*.

In our study, single-copy CDS plastome sequences produced a robust phylogeny for *Kitagawia*. In the CDS tree, all six *Kitagawia* species examined fell into Selineae and were divided into two branches (Subclade I and Subclade II) (Figure 1). They were all considerably distant from *P. morisonii*, the member of *Peucedanum* sensu stricto confirmed in previous studies [2,8,29] (Figure 1). Similar results were presented in the nrDNA (ITS + ETS) tree (Figure 1). *Peucedanum officinale* L., the type species of *Peucedanum* sensu stricto, formed a sister branch to *P. morisonii*, which was closely related to four *Angelica* species and more distantly related to the six *Kitagawia* species examined (Figure 1). In both phylogenetic trees, Subclade I and *C. sinensis*, and Subclade II and *S. divaricata* formed sister branches, respectively (Figure 1). Although there were differences in the topologies of plastome CDS and nrDNA (ITS + ETS) trees, they consistently indicated considerably distant phylogenetic relationships between *Kitagawia* and *Peucedanum* sensu stricto, thus providing strong support for the separation of *Kitagawia*. These trees also revealed that *Kitagawia*, as defined by Pimenov, was not monophyletic [4,10,17,18].

We believe that the “core” *Kitagawia* should be limited to Subclade I, while *K. praeruptora* and *K. formosana* should be removed from the genus. Furthermore, *P. hakuunense* was treated as a synonym of *K. komarovii* by Pimenov [18]; while our results showed that *P. hakuunense* should be identified as a distinct species, both *P. hakuunense* and *P. chujaense* may belong to *Kitagawia*. Our phylogenetic analyses confirmed that *Kitagawia* does not belong to the *Acronema* Clade but to Selineae. There was no doubt that some accessions of *Kitagawia* used in previous studies were misidentified [27,29,30]. Our results demonstrate the rationale behind the separation of *Kitagawia* and the necessity for its further taxonomic revision.

### 3.2. Plastome Structure and Evolution and Their Phylogenetic Implications

Our comparative analyses revealed that all 14 plastomes examined displayed a typical quadripartite structure [43,47] (Figure S1). The plastome structure, GC content, and gene order were similar to those of species in the Apiaceae tribe Selineae (Figures S1 and S2, Table 1), implying that plastome structure was highly conserved [43,47,55]. Among the 14 species examined, the plastomes of *K. praeruptora*, *K. formosana,* and *P. harry-smithii* var. *grande* lacked the *trnT-GGU* gene (Table S2), which may suggest that they have different evolutionary histories when compared to other plastomes [56,57].

SSRs have been widely used as molecular markers in plant population genetics and evolutionary studies [58,59,60]. In the 14 studied plastomes of *Kitagawia* and related taxa, mononucleotides were the most abundant SSRs, followed by dinucleotides, tetranucleotides, trinucleotides, pentanucleotides, and hexanucleotides. Such findings are widespread in Apiaceae, Liliaceae, and *Allium* L. [47,61,62]. All identified SSRs were mainly composed of bases A and T, causing an AT richness in the overall plastome [43,63]. IR expansion and contraction have been recognized as evolutionary markers for illustrating relationships among taxa [43,55,64]. In this study, the JLA of species within Subclade I and Subclade II visibly differed from other species examined (Figure 3). Distances between gene *trnL* and JLA of species within Subclade I and Subclade II were considerably longer than *P. morisonii*, *C. sinensis*, and *A. sylvestris* (Figure 3). In contrast, distances from gene *trnH* to JLA of species within Subclade I and Subclade II were shorter than for the three above-mentioned species (Figure 3). Furthermore, the lengths of the IR regions of *P. morisonii*, *C. sinensis*, and *A. sylvestris* were shorter than for other species examined (18,457–19,043 bp) (Figure 3, Table 1). The similar features of JLA and the IR length of *P. morisonii*, *C. sinensis*, and *A. sylvestris* may imply a common but different evolutionary history that differentiated them from the others (Subclade I and II and *S. divaricata*). In addition, sequence divergences noted in several regions (e.g., *ycf1*, *ycf2*, and *accD-psaI*) separated Subclade I from Subclade II and *P. morisonii* (Figure 4). These differences in plastomes may reflect distinct phylogenetic affinities between the species of Subclade I and the others [55]. Overall, the results of comparative plastome analyses revealed different evolutionary relationships of Subclade I and Subclade II to other taxa examined and added some support to the distinct phylogenetic position and non-monophyly of *Kitagawia*.

### 3.3. Morphological Delimitations between Kitagawia and Related Taxa

In this study, we examined fruit anatomical and micromorphological characteristics of five *Kitagawia* species (i.e., *K. baicalensis*, *K. komarovii*, *K. praeruptora*, *K. stepposa*, and *K. terebinthacea*). The results showed that all species shared filiform dorsal ribs, winged marginal ribs, flat or slightly concave endosperm commissural face, partly lignified parenchyma cells with pitted walls in marginal ribs, inconspicuous cell borders of fruit surfaces, without epicuticular wax (Figure 5 and Figure 6, Table 2). These findings support the recognition of *Kitagawia* at a generic level [10]. However, compared with the four “core” *Kitagawia* species within Subclade I (*K. baicalensis*, *K. komarovii*, *K. stepposa*, and *K. terebinthacea*), *K. praeruptora* within Subclade II had more vallecular vittae (3–4 vs. solitary) and commissural vittae (6 vs. 2), more layers of mesocarp cells closest to the epidermis (3–5 vs. 1–2), and narrower marginal ribs and commissure (Figure 5, Table 2). These differences run counter to the descriptions of *Kitagawia* [4,10,17], implying that *K. praeruptora* should be removed from *Kitagawia*. Furthermore, the five *Kitagawia* species examined had different fruit surfaces and various cuticular foldings (Figure 6, Table 2). These characteristics partly overlapped with the fruit micromorphological characteristics of *Peucedanum* sensu stricto [23,25,65] and were incongruent with the results of Ostroumova [23,24,25]. Thus, micromorphological characteristics of fruit surfaces might vary among populations, which would make them poor diagnostic characteristics for *Kitagawia*.

Furthermore, the four “core” *Kitagawia* species within Subclade I differed from *C. sinensis* in bracts and bracteoles (linear or lanceolate vs. linear), mericarp shape (elliptic vs. oblong-ovate), mericarp surfaces (glabrous vs. densely hirtellous), and marginal rib shape of mericarps (winged vs. filiform) [66] (Table S3). Similarly, they were distinct from *A. sylvestris*, which has linear bracts and bracteoles, pubescent rays, obsolete calyx teeth, broadly ovate mericarps, and narrowly winged and obtuse dorsal ribs [17,67] (Table S3). It is notable that the four “core” *Kitagawia* species within Subclade I were slightly different from the representative members of *Peucedanum* sensu stricto (*P. morisonii* and *P. officinale*) in leaf blades (2–3-pinnate vs. 3–6-ternate), bracts (lanceolate vs. subulate to linear), bracteoles (lanceolate vs. linear), rays (adaxial surface hispid vs. glabrous), and petals (white vs. yellow) [11,12,68] (Table S3). These partially overlapping traits reflect the difficulties in dividing *Peucedanum* sensu lato and indicate that it is ill-advised to distinguish *Kitagawia* from *Peucedanum* sensu stricto by individual morphological characteristics.

The association of a series of morphological characteristics is regarded as a more powerful tool to distinguish taxonomically difficult taxa compared to a single morphological characteristic [55]. Through our analyses, the morphological delimitation of *Kitagawia* (the four “core” *Kitagawia* species within Subclade I) should be “stems glabrous, leaf blades 2–3-pinnate, ultimate leaf segments linear to lanceolate, bracts and bracteoles lanceolate, rays adaxial surface hispid, petals white, calyx teeth subulate or triangular, stylopodia conical, mericarps strongly compressed dorsally, elliptic and glabrous, dorsal ribs filiform and slightly prominent, marginal ribs winged, commissure broad, solitary vitta in each furrow, two vittae on commissure, partly lignified parenchyma cells with pitted walls in marginal ribs, 1–2 layers of mesocarp cells closest to the epidermis, inconspicuous cell borders of fruit surfaces, without epicuticular wax”. Considering that *P. hakuunense* and *P. chujaense* probably belong to *Kitagawia*, the morphological delimitation of *Kitagawia* may need further revision.

### 3.4. Taxonomic Suggestions for Six Species from Kitagawia and Peucedanum Sensu Lato

Pimenov stated that the generic boundaries of *Kitagawia* might need to be expanded and highlighted several *Peucedanum* sensu lato species that may belong to *Kitagawia* [10,17]. One example was discussed in the taxonomic treatment of *K. formosana* [18].

In this study, two *Peucedanum* sensu lato species considered as candidates for *Kitagawia*, *P. ampliatum* and *P. harry-smithii* var. *grande*, were included to investigate the generic boundaries of *Kitagawia*. In our phylogenetic trees, *P. ampliatum* and *P. harry-smithii* var. *grande* clustered with *K. praeruptora* and *K. formosana*, forming Subclade II (Figure 1). The phylogenetic positions of the four species within Subclade II were distant from Subclade I (“core” *Kitagawia*) and *Peucedanum* sensu stricto (Figure 1). Instead, they are closely related to *S. divaricata*, the type species of a monotypic genus *Saposhnikovia* (Figure 1). The rather distant relationship of Subclade II to Subclade I and *Peucedanum* sensu stricto suggest that the four species within Subclade II (including *K. praeruptora* and *K. formosana*) should neither be considered members of *Kitagawia* nor included in *Peucedanum* sensu lato, which is consistent with previous phylogenetic results [32,33,47,69].

In our morphological analyses, the four species within Subclade II, *K. praeruptora*, *K. formosana*, *P. ampliatum,* and *P. harry-smithii* var. *grande*, shared characteristics “stems pubescent or tomentose, bracts absent or few, linear or lanceolate, bracteoles numerous, linear or lanceolate, rays pubescent or tomentose on adaxial surface or throughout, petals white, mericarps pubescent or hispid, dorsal ribs filiform and prominent, marginal ribs narrowly winged, 3–5 vittae in each furrow, 6–8 vittae on commissure” [11,12,70], which differed from the four *Kitagawia* species within Subclade I, supporting the distant phylogenetic relationship of *K. praeruptora* and *K. formosana* to “core” *Kitagawia* (Figure 1, Table S3). These characteristics shared by the four species within Subclade II could also distinguish them from *Peucedanum* sensu stricto [4] and *Saposhnikovia* (the latter have glabrous stems, rays, and mericarp, few bracteoles, solitary vitta in each furrow, two vittae on commissure, and one pseudovitta under each primary rib) [71] (Table S3). As far as the present study is concerned, the members of Subclade II possessed unique fruit anatomical and micromorphological characteristics, as well as general morphological characteristics, that could not be identified in other related genera (Tables S2 and S3, Figure 5 and Figure 6). This may suggest that their current taxonomic placements are unreasonable. The phylogenetic and morphological results consistently suggested that *K. praeruptora* and *K. formosana* should be placed in a new genus and the two possible candidate taxa (*P. ampliatum* and *P. harry-smithii* var. *grande*) should not be included in *Kitagawia*. For the possible new genus, more extensive population sampling and study of additional, potentially related taxa are required to determine generic boundaries and morphological delimitations.

Two *Peucedanum* sensu lato species found in Korea, *P. hakuunense* and *P. chujaense*, fell into Subclade I. Of these, *P. hakuunense* was treated as a synonym of *K. komarovii* [18]. *Peucedanum chujaense* K. Kim, S.H. Oh, Chan S. Kim & C.W. Park was thought to be morphologically most similar to *Kitagawia* species [15]. Our phylogenetic analyses revealed that *P. hakuunense* and *P. chujaense* nested into Subclade I, suggesting they may need to be included in *Kitagawia* (Figure 1). However, specimens of *P. hakuunense* and *P. chujaense* were not available and morphological evaluations could not be undertaken. In our morphological analyses, therefore, the general morphological characteristics of *P. hakuunense* and *P. chujaense* were directly referenced from previous literature [15,72,73] to document the morphological similarity of these two species with the four *Kitagawia* species within Subclade I (Table S3). Phylogenetic and morphological evidence suggests that *P. hakuunense* and *P. chujaense* probably belong to *Kitagawia*. More evidence is needed for a complete taxonomic treatment of *P. hakuunense* and *P. chujaense* and for the clarification of interspecific relationships within Subclade I. To that end, international cooperation will be needed to produce a more detailed revision of *Kitagawia*.

### 3.5. Topologic Incongruence between Phylogenies based on Plastome and NrDNA Sequences

Previous molecular phylogenetic studies have identified a common phylogenetic incongruence between plastid and nuclear gene trees [43,74]. Several evolutionary processes, such as hybridization, introgression, and incomplete lineage sorting (ILS), are regarded as plausible explanations for the discordance of plastid and nuclear DNA phylogenies [75,76].

In our phylogenetic analyses, the phylogenetic positions of three main clades (i.e., Tordyliinae, Coriandreae, and *Hymenidium* Clade) differed between the two phylogenetic trees (Figure 2). These differences were also reported by Wen et al. [46]. We agree with Wen et al. [46] that the conflicting positions of the three main clades (Tordyliinae, Coriandreae, and *Hymenidium* Clade) between the plastome CDS and nrDNA (ITS + ETS) trees were mainly caused by chloroplast capture. In Selineae, some branches (e.g., the branch Subclade I + *C. sinensis* and the branch Subclade II + *S. divaricata*) and even some species within the same branch differed in their phylogenetic placements in the phylogenies based on plastome CDS and nrDNA (ITS + ETS) sequences (Figure 1). Similar to the three main clades (Tordyliinae, Coriandreae, and *Hymenidium* Clade), the incongruent positions of the four branches (i.e., “*Peucedanum* sensu stricto group”, “*Angelica* group”, Subclade I + *Carlesia*, and Subclade II + *Saposhnikovia*) between the plastome CDS and nrDNA (ITS + ETS) trees may also have been generated by early chloroplast capture events due to hybridization. Our phylogenetic results supported a chloroplast capture hypothesis that the early lineage of the branch Subclade I + *Carlesia* may have successively captured the chloroplast genome of ancestors of the “*Angelica* group”. The strongly supported polytomy of Subclade II in the nrDNA (ITS + ETS) tree suggested the insufficiency of phylogenetic informative characters in the nrDNA dataset, which is also thought to contribute to the incongruent topologies between plastid and nuclear gene trees [77,78]. The lack of informative characters is usually derived from rapid radiation and hybridization [79,80]. In our study, hybridization is not a reasonable explanation for the polytomy of Subclade II because the four species within Subclade II included two stenochoric species (*K. formosana* and *P. ampliatum*) with non-overlapping distributions [12,70]. Rapid radiation may be the most likely mechanism for the polytomy of Subclade II in the nrDNA (ITS + ETS) tree. The hairy stems and fruits and numerous mericarp vittae may be synapomorphies of the four species within Subclade II (Table S4), and they may have undergone rapid morphological differentiation by a founder effect [81], which can coincide with rapid radiation.

## 4. Materials and Methods

### 4.1. Taxon Sampling

Fresh green leaves from adult plants of seven taxa, including five *Kitagawia* species (*K. baicalensis*, *K. komarovii*, *K. praeruptora*, *K. stepposa,* and *K. terebinthacea*), *C. sinensis*, and *S. divaricata* were collected from the wild, and then immediately dried with silica gel to preserve them for DNA extraction. Special permission was not required to collect these materials because they are not key-protected plants. In addition, we requested and obtained the genomic DNA of *K. formosana* from the Herbarium of the Institute of Botany (PE), Chinese Academy of Sciences. The formal identification of all samples was undertaken by Professor Xingjin He (Sichuan University). The voucher information of the eight samples is summarized in Table S4.

### 4.2. DNA Extraction, Sequencing, Assembly, and Annotation

The total genomic DNA of the eight taxa was extracted from silica gel-dried leaves or herbarium specimens with a modified CTAB protocol [82]. The primers ITS4 (5′-TCC TCC GCT TAT TGA TAT GC-3′) and ITS5 (5′-GGA AGT AAA AGT CGT AAC AAG G-3′) were used in the polymerase chain reaction (PCR) amplification of the complete ITS region [83]. The ETS sequences were amplified with primers 18S-ETS (5′-ACT TAC ACA TGC ATG GCT TAA TCT-3′) and Umb-ETS (5′-GCG CAT GAG TGG TGA WTK GTA-3′) [84,85]. The 30-µL PCR reactions contained 2 µL extracted total genomic DNA, 10 µL ddH_2_O, 1.5 µL each of 10 pmol µL^−1^ forward and reverse primers, and 15 µL Taq MasterMix (CWBio, Beijing, China). The PCR program of ITS and ETS regions started with an initial denaturation at 94 °C for 4 min, followed by 30 cycles of denaturation at 94 °C for 45 s, annealing at 54 °C for 45 s, and extension at 72 °C for 1 min, with a final extension at 72 °C for 10 min. All PCR products were separated on a 1.5% (w v^−1^) agarose TAE gel and sent to Sangon (Shanghai, China) for sequencing. The newly sequenced ITS and ETS sequences of the eight taxa were examined and edited with Geneious v9.0.2 [86], and consensus sequences were obtained separately.

Plastomes of the eight taxa, namely *K. baicalensis*, *K. formosana*, *K. komarovii*, *K. praeruptora*, *K. stepposa*, *K. terebinthacea*, *C. sinensis*, and *S. divaricata*, were sequenced. We provided 20 µL of total genomic DNA per species for the sequencing process, and total genomic DNA was sequenced on an Illumina HiSeq X Ten platform (paired-end, 150 bp) by Novogene (Beijing, China). At least 5 GB of raw data per species were generated. Quality control of the raw reads was performed using fastP v0.15.0 (-n 10 and -q 15) [87], yielding high-quality reads for each species. The program GetOrganelle v1.7.5.3 [88] was used to assemble the complete plastomes with the custom parameters (-F embplant_pt; -R 15; -k 21, 45, 65, 85, 105, 121). Assembled plastomes of the eight taxa were annotated with PGA [89]. Geneious v9.0.2 [86] was then used to manually adjust the annotation for uncertain start and stop codons based on comparisons with homologous genes from other annotated plastomes. The circular plastome maps were generated with OGDRAW v1.3.1 [90].

The newly obtained complete plastomes, ITS, and ETS sequences of the eight taxa studied were submitted to GenBank under accession numbers OP379697-OP379704, OP377020-OP377027, and OP379688-OP379695, respectively (Table S5).

### 4.3. Phylogenetic Analyses

After preliminary analyses, 28 species from Selineae, Tordyliinae, Coriandreae, *Hymenidium* Clade, Apieae, Careae, Pyramidoptereae, and *Acronema* Clade were selected to conduct the phylogenetic reconstruction. The type species of *Peucedanum* sensu stricto and *Angelica*, *P. officinale* and *A. sylvestris*, were included due to the blurred boundaries and potential expansion of *Kitagawia* [10]. Similarly, *P. ampliatum* and *P. harry-smithii* var. *grande* were added to examine the possible candidates for *Kitagawia* according to Pimenov and Ostroumova [17]. *Pternopetalum davidii* Franch. and *Pternopetalum vulgare* (Dunn) Hand.-Mazz., which belong to *Acronema* Clade, were chosen as the outgroup to root the phylogenetic tree, based on the results of Downie et al. [30]. The names of the main clades refer to the contributions of Downie et al. [30] and Gou et al. [51].

Two datasets were constructed for the phylogenetic analyses. The CDS dataset consisted of single-copy CDS sequences from complete plastomes of 27 taxa (6 species from *Kitagawia* and 21 species from other genera in the above-mentioned clades). To provide greater branch support [85], the nrDNA dataset concatenated the complete ITS and ETS regions from 27 taxa of the same genera used in the CDS dataset. *Peucedanum officinale* L. and *P. chujaense* could not be added to the CDS dataset and nrDNA dataset, respectively, due to a lack of online data. All sequences used in phylogenetic analyses are available in GenBank (Table S5).

The 79 single-copy CDS sequences from 27 complete plastomes were extracted and connected using PhyloSuite v1.2.2 [91]. The nrDNA (ITS + ETS) sequences of 27 taxa were also concatenated with PhyloSuite v1.2.2 [91]. Sequences of the two datasets were aligned with MAFFT v7.221 [92] and then manually corrected with MEGA7 [93]. Maximum likelihood (ML) and Bayesian inference (BI) methods were adopted to infer phylogenetic relationships. RAxML v8.2.10 [94] was used to perform the ML analyses for the two datasets based on the GTRGAMMA model and 1000 rapid bootstrap replicates. MrBayes v3.2.7 [95] was used to perform the BI analyses with the best substitution model as determined by MrModeltest v2.4 [96]. The selected models for the CDS dataset and nrDNA dataset in BI analyses were GTR + I + G and GTR + G, respectively. Four independent Markov chains were run for 10,000,000 generations with random initial trees, sampling every 1000 generations. The first 25% of trees were discarded as burn-in, and the remaining trees were used to build a 50% majority-rule consensus tree. The phylogenetic trees were visualized and edited with FigTree v1.4.4 [97] and MEGA7 [93].

### 4.4. Comparative Analyses of Plastomes

To better understand the genomics and evolution of *Kitagawia* plastomes, 14 plastomes representing *Kitagawia* and related taxa were selected for comparative analyses of plastomes based on our phylogenetic results. They consisted of six *Kitagawia* plastomes (*K. baicalensis*, *K. formosana*, *K. komarovii*, *K. praeruptora*, *K. stepposa*, and *K. terebinthacea*), five *Peucedanum* sensu lato plastomes (*P. ampliatum*, *P. chujaense*, *P. hakuunense*, *P. harry-smithii* var. *grande*, and *P. morisonii*), *A. sylvestris*, *C. sinensis*, and *S. divaricata*.

Simple sequence repeats (SSRs) for each plastome were detected with MISA (http://pgrc.ipk-gatersleben.de/misa/ (accessed on 21 May 2022)) [98]. The minimum numbers of the SSRs were set as 10, 5, 4, 3, 3, and 3 for mono-, di-, tri-, tetra-, penta-, and hexa-nucleotides, respectively.

The expansion or contraction between IR border regions of the 14 plastomes of *Kitagawia* and related taxa were drawn by IRscope [99] and adjusted manually.

The DNA rearrangements among the 14 selected plastomes were detected using Mauve Aligner [100] implemented in Geneious v9.0.2 [86]. Sequence divergence of the 14 complete plastomes was visualized with mVISTA [101] under Shuffle-LAGAN alignment mode [102] with *K. terebinthacea* as the reference.

### 4.5. Morphological Analyses

#### 4.5.1. Fruit Anatomical and Micromorphological Characteristics

Mature fruits of five *Kitagawia* species (*K. baicalensis*, *K. komarovii*, *K. praeruptora*, *K. stepposa*, and *K. terebinthacea*) were collected from the wild and preserved in formaldehyde–acetic acid–alcohol (FAA) for morphological examination. Fruit anatomy was evaluated by paraffin section. For each of the five species, the fruits of at least three individuals from the same population were examined. All fruits were treated following the method of Feder and O’Brien [103] for embedding in glycol methacrylate (GMA). A Leica RM2016 microtome was used to prepare transverse sections through the center of the mericarp, about 3–5 mm in thickness, and they were stained with toluidine blue. Sections were observed and photographed under an Olympus BX43 microscope. Morphological terminology followed Kljuykov et al. [104].

Fruit micromorphology was examined by scanning electron microscope (SEM). After dehydration using graded ethanol, all mature fruits were directly mounted on clean aluminum stubs with conducting carbon adhesive tabs, coated, and then scanned with a JSM-7500F scanning electron microscope. The micromorphological characteristics studied and micromorphological terminology follow the contributions of Ostroumova [23,24].

#### 4.5.2. General Morphological Characteristics

We examined the general morphological characteristics of the 14 species used in the above comparative plastome analyses together with *P. officinale*, the type species of *Peucedanum* sensu stricto. The characteristics examined included features of the stem, leaf, bract, bracteole, ray, flower, and fruit. Data from the five *Kitagawia* species (*K. baicalensis*, *K. komarovii*, *K. praeruptora*, *K. stepposa*, and *K. terebinthacea*) were obtained mainly from our field-based observations and anatomy research. For some taxa and characteristics, data were obtained directly from previous literature [11,12,15,17,66,67,68,71,72,73].

## 5. Conclusions

In this study, we newly sequenced complete plastomes of eight species from *Kitagawia*, *Carlesia*, and *Saposhnikovia*. The phylogeny reconstruction of *Kitagawia* was performed based on plastome CDS and nrDNA (ITS + ETS) sequences. Our results revealed that *Kitagawia* is a member of the tribe Selineae but is divided into two branches (Subclade I and Subclade II). The distant phylogenetic relationships between *Kitagawia* and *Peucedanum* sensu stricto verified the rationale behind the separation of *Kitagawia*. The visible differences in JLA and several highly variable regions added some support to the distinct phylogenetic position and non-monophyly of *Kitagawia*. Similarly, fruit anatomical and micromorphological characteristics, as well as general morphological characteristics, distinguished Subclade I from Subclade II and other related taxa, further supporting the results of phylogenetic analyses. These findings demonstrated that *P. ampliatum* and *P. harry-smithii* var. *grande* should not be included in *Kitagawia* and suggested that *K. praeruptora* and *K. formosana* should be placed in a new genus and that *P. hakuunense* and *P. chujaense* probably belong to *Kitagawia*. We believe that the “core” *Kitagawia* should be limited to Subclade I, and this genus can be distinguished by the association of a series of morphological characteristics. In short, our study confirmed the distinct generic status of *Kitagawia* and clarified the morphological delimitations between *Kitagawia* and related taxa, providing a foundation for further taxonomic and evolutionary research on *Kitagawia*.

## Figures and Tables

**Table 1:** Features of the 14 plastid genomes of *Kitagawia* and related taxa.

Taxa	Length (bp)				Number of Unique Genes				GC Content (%)			
	Genome	LSC	SSC	IR	Total	PCG	tRNA	rRNA	Total	LSC	SSC	IR
*K. baicalensis*	147,985	93,396	17,619	18,485	114	80	30	4	37.5	35.9	31.0	44.6
*K. formosana*	146,718	91,829	17,581	18,654	113	80	29	4	37.6	36.1	31.0	44.5
*K. komarovii*	147,790	93,225	17,631	18,467	114	80	30	4	37.5	35.9	31.1	44.6
*K. praeruptora*	147,035	92,072	17,535	18,714	113	80	29	4	37.6	36.1	31.1	44.5
*K. stepposa*	147,965	93,376	17,619	18,485	114	80	30	4	37.5	36.0	31.0	44.6
*K. terebinthacea*	148,327	93,078	17,605	18,822	114	80	30	4	37.5	35.9	30.9	44.3
*P. ampliatum*	147,403	92,526	17,519	18,679	114	80	30	4	37.6	36.0	31.0	44.5
*P. chujaense*	147,839	93,335	17,590	18,457	114	80	30	4	37.4	35.9	30.8	44.6
*P. hakuunense*	147,426	91,915	17,425	19,043	114	80	30	4	37.5	35.9	30.9	44.4
*P. harry-smithii* var. *grande*	147,046	92,135	17,527	18,692	113	80	29	4	37.6	36.0	31.1	44.5
*P. morisonii*	147,105	93,594	17,537	17,987	114	80	30	4	37.6	36.1	31.1	44.8
*A. sylvestris*	147,158	93,479	17,563	18,058	114	80	30	4	37.5	35.9	31.0	44.8
*C. sinensis*	147,436	93,700	17,546	18,095	114	80	30	4	37.5	35.9	31.0	44.7
*S. divaricata*	147,938	93,233	17,377	18,664	114	80	30	4	37.5	35.9	30.8	44.6

**Table 2:** Fruit anatomical and micromorphological characteristics of five *Kitagawia* species.

Taxa	*K. baicalensis*	*K. stepposa*	*K. komarovii*	*K. terebinthacea*	*K. praeruptora*
Mericarps	Strongly compressed dorsally	Strongly compressed dorsally	Strongly compressed dorsally	Strongly compressed dorsally	Slightly compressed dorsally
Dorsal rib shape	Filiform, slightly prominent	Filiform, slightly prominent	Filiform, slightly prominent	Filiform, slightly prominent	Filiform, prominent
Marginal rib shape	Winged	Winged	Winged	Winged	Narrowly winged
Vallecular vittae	1	1	1	1	3–4
Commissural vittae	2	2	2	2	6
Endosperm	Slightly concave	Slightly concave	Flat	Flat	Flat
Layers of mesocarp cells	1–2	1–2	1–2	1–2	3–5
Mesocarp parenchyma	Lignified parenchyma with pitted walls	Lignified parenchyma with pitted walls	Lignified parenchyma with pitted walls	Lignified parenchyma with pitted walls	Lignified parenchyma with pitted walls
Average of marw/fw (%)	42.3	48.8	44.6	50.3	39.8
Average of cow/fw (%)	96.4	94.0	95.4	94.1	85.2
Cell borders (outer surface of fruits)	Inconspicuous	Inconspicuous	Inconspicuous	Inconspicuous	Inconspicuous
Fruit surfaces	Undulate	Rugate	Tuberculate	Undulate	Raised
Cuticular foldings	Striate and rugulate	Smooth and striate	Smooth and striate	Striate and rugulate	Rugulate and tuberculate
Epicuticular wax	Absent	Absent	Absent	Absent	Absent
Inner fruit structure	Parenchyma cells with pitted walls	Parenchyma cells with pitted walls	Parenchyma cells with pitted walls	Parenchyma cells with pitted walls	Parenchyma cells with pitted walls

Note: marw/fw: the ratio of the total widths of both marginal ribs to the fruit width; cow/fw: the ratio of the commissure width to the fruit width.

## Data Availability

The annotated plastomes, ITS, and ETS sequences of the eight taxa studied have been submitted to NCBI (https://www.ncbi.nlm.nih.gov (accessed on 7 September 2022)) with accession numbers that can be found in Table S5.

## Supplementary Materials

The following supporting information can be downloaded at: https://www.mdpi.com/article/10.3390/plants11233275/s1, Figure S1: Gene maps of the eight plastomes of *Kitagawia*, *Carlesia*, and *Saposhnikovia*; Table S1: List of genes identified in the 14 plastomes of *Kitagawia* and related taxa; Table S2: Number of all identified SSRs in the 14 plastomes; Figure S2: Mauve alignment of the 14 plastomes of *Kitagawia* and related taxa; Table S3: Synopsis of general morphological characteristics from the 15 species involved in this study; Table S4: Collection locality and voucher information are provided for the eight sequenced plastomes; Table S5: GenBank accession numbers of DNA sequences used in this study.
