# Supplementary material for: Evidence from Phylogenomics and Morphology Provide Insights into the Phylogeny, Plastome Evolution, and Taxonomy of Kitagawia
Source: Plants (Basel). 2022 Nov 28;11(23):3275. doi: 10.3390/plants11233275 (PMC9740501; doi:10.3390/plants11233275)
Supplement: Supplementary file 1 [file plants-11-03275-s001.zip › Figure S1.pdf]

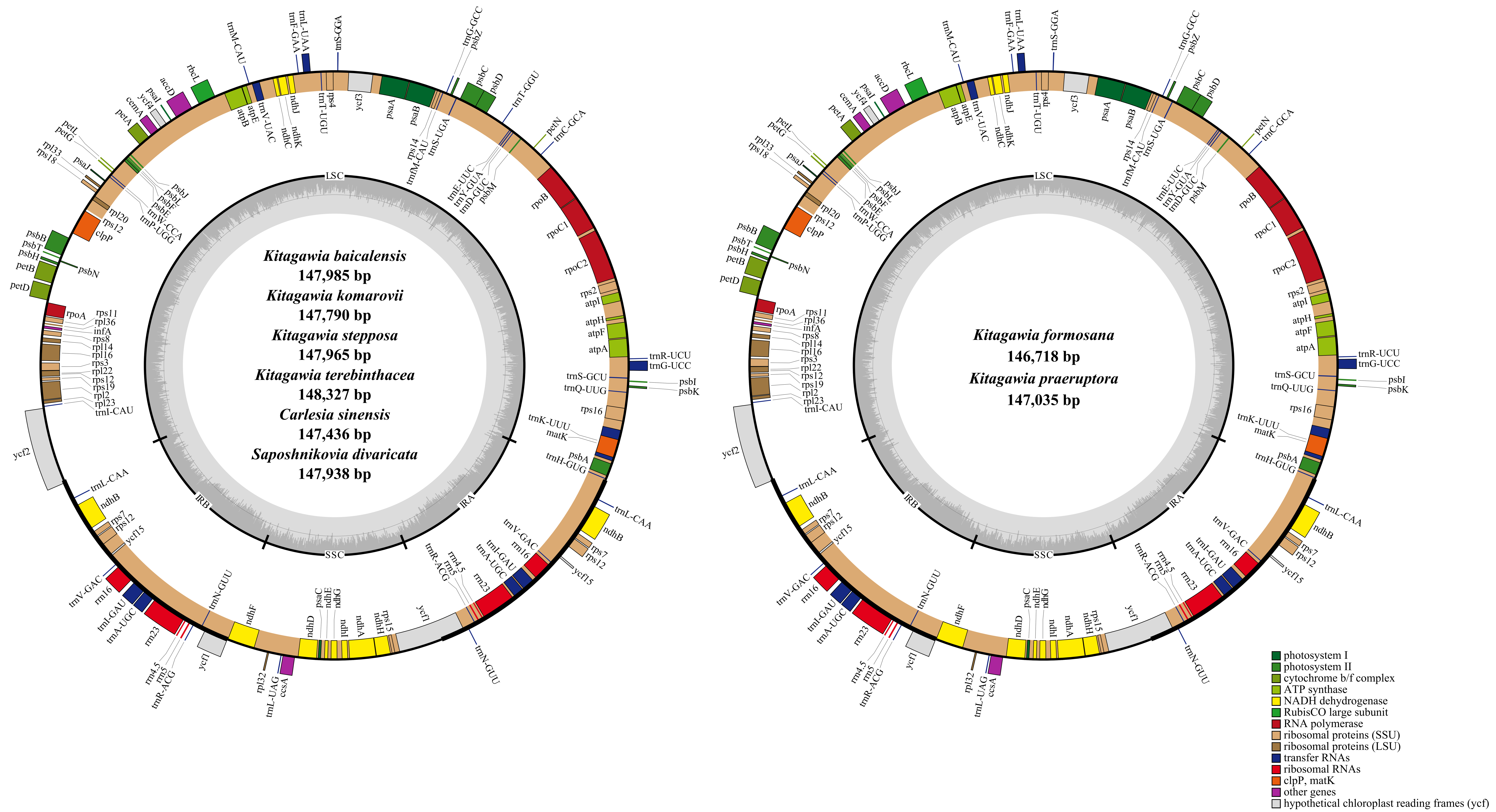

Figure S1. Gene maps of the eight plastomes of *Kitagawia*, *Carlesia*, and *Saposhnikovia*. Genes shown inside and outside the outer layer circle are transcribed in the clockwise and counterclockwise, respectively. Different functional groups of genes are marked with different colors. The dark gray area of the inner circle represents the GC content of the plastome.
