# Supplementary material for: Evidence from Phylogenomics and Morphology Provide Insights into the Phylogeny, Plastome Evolution, and Taxonomy of Kitagawia
Source: Plants (Basel). 2022 Nov 28;11(23):3275. doi: 10.3390/plants11233275 (PMC9740501; doi:10.3390/plants11233275)
Supplement: Supplementary file 1 [file plants-11-03275-s001.zip › Figure S2.pdf]

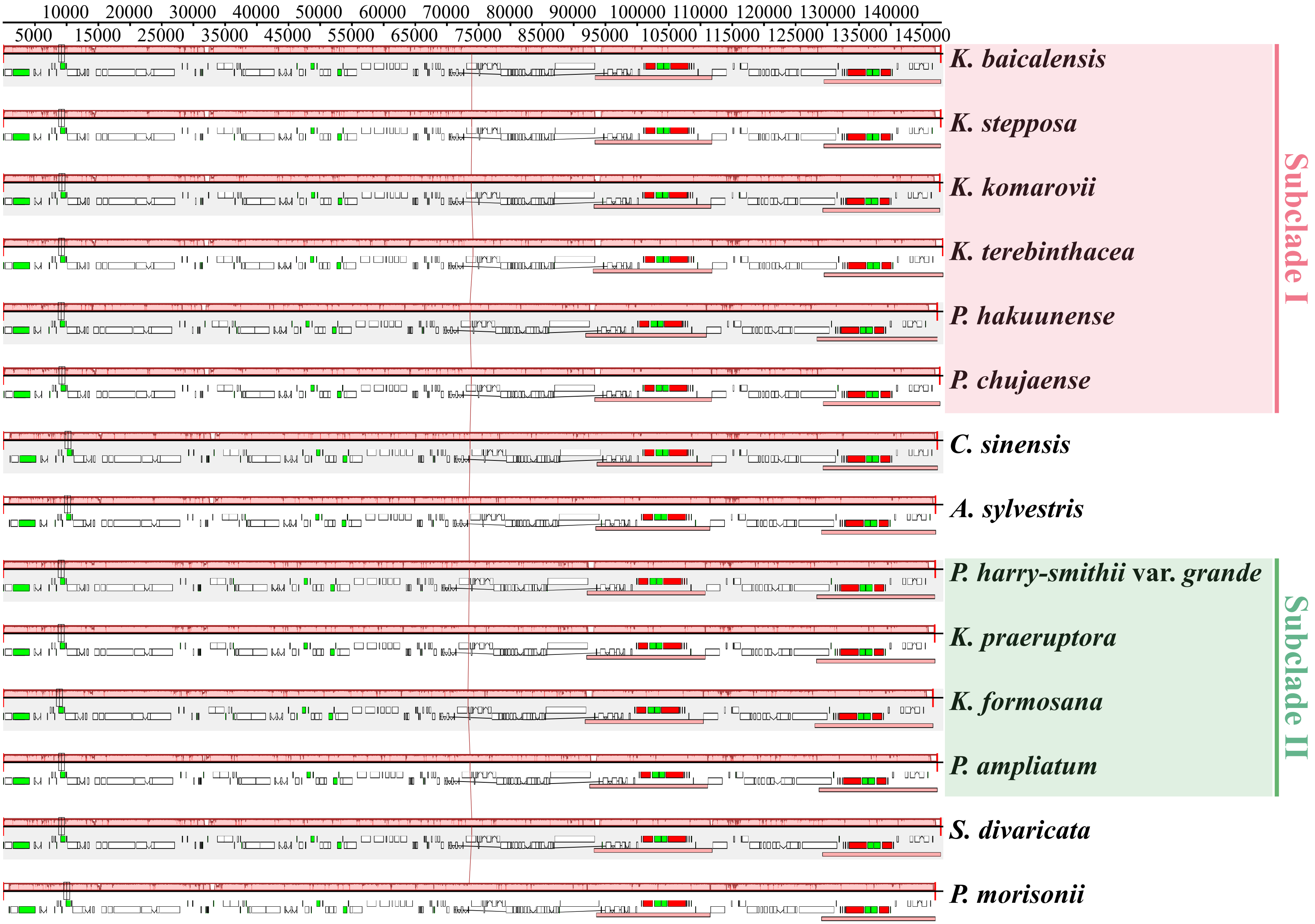

Figure S2. Mauve alignment of the 14 plastomes of *Kitagawia* and related taxa. Locally collinear block (LCB) within each alignment is represented by block of the same color connected with line.
